# Large-scale synthesis of uniform hexagonal boron nitride films by plasma-enhanced atomic layer deposition

**DOI:** 10.1038/srep40091

**Published:** 2017-01-05

**Authors:** Hamin Park, Tae Keun Kim, Sung Woo Cho, Hong Seok Jang, Sang Ick Lee, Sung-Yool Choi

**Affiliations:** 1 School of Electrical Engineering, Center for Advanced Materials Discovery for 3D Display, Graphene/2D Materials Research Center, KAIST, Daejeon 34141, Republic of Korea; 2 DNF Co., Ltd., Daejeon 34366, Republic of Korea

## Abstract

Hexagonal boron nitride (h-BN) has been previously manufactured using mechanical exfoliation and chemical vapor deposition methods, which make the large-scale synthesis of uniform h-BN very challenging. In this study, we produced highly uniform and scalable h-BN films by plasma-enhanced atomic layer deposition, which were characterized by various techniques including atomic force microscopy, transmission electron microscopy, Raman spectroscopy, and X-ray diffraction. The film composition studied by X-ray photoelectron spectroscopy and Auger electron spectroscopy corresponded to a B:N stoichiometric ratio close to 1:1, and the band-gap value (5.65 eV) obtained by electron energy loss spectroscopy was consistent with the dielectric properties. The h-BN-containing capacitors were characterized by highly uniform properties, a reasonable dielectric constant (3), and low leakage current density, while graphene on h-BN substrates exhibited enhanced electrical performance such as the high carrier mobility and neutral Dirac voltage, which resulted from the low density of charged impurities on the h-BN surface.

In the past ten years, two-dimensional (2-D) materials have attracted a lot of interest because of their ability to exhibit conducting, semiconducting, or insulating characteristics depending on the composition and atomic structure. Hexagonal boron nitride (h-BN) has been most widely investigated among the insulating 2-D materials and is characterized by a wide band-gap (5.5–6.0 eV) and inherently flat surface that does not contain dangling bonds or charged impurities, which makes it potentially suitable for dielectric layers in electronic devices.

Similar to other 2-D materials, h-BN has been previously produced by mechanical exfoliation method and exhibited high performance in electronic and optoelectronic applications^1^,^2^. However, the scalability of the mechanical exfoliation method is very poor. Recently, several researchers have reported the chemical vapor deposition (CVD) of h-BN films on various metal substrates including Cu^3^, Ni^4^, Fe^5^, and Pt^6^ (ammonia borane^3^ or borazine^4^,^5^,^6^ was used as precursor). Also, direct h-BN synthesis via oxygen-assisted CVD on dielectric substrate has reported for eliminating the need for metal catalyst and transfer process^7^. CVD enables the large-scale synthesis of h-BN films, however, CVD-grown h-BN layers exhibited highly non-uniform properties across the film surface, such as wide thickness and dielectric constant distributions. Furthermore, the CVD method requires high temperatures (around 1,000 °C) and transfer process from metal foil to target substrates for various applications.

Atomic layer deposition (ALD) is a thin film deposition technique that is capable of producing uniform large-scale films, which are characterized by conformality to high aspect ratio structures, and highly controllable thicknesses at relatively low temperatures. ALD is widely used to deposit dielectric materials such as HfO_2_ and Al_2_O_3_ due to its advantages over the CVD or physical vapor deposition methods. Recently, it has been applied to synthesize 2-D materials such as MoS_2_^8^ and WS_2_^9^ which exhibit semiconducting characteristic. However, ALD is not often utilized for manufacturing 2-D dielectric materials such as h-BN films. Although several studies reported the synthesis of BN by ALD, they did not include the investigation of its atomic-scale properties or electrical performance^10^,^11^,^12^.

In this work, we describe the large-scale synthesis of uniform h-BN films by plasma-enhanced atomic layer deposition (PE-ALD), which are characterized by highly flat surfaces, high uniformity, and controllable thickness at low temperatures (250–350 °C). The morphology and composition of the obtained films were investigated by various optical and spectroscopic analytical methods, and their ability to be potentially used as substrates for graphene field-effect transistors (GFETs) has been demonstrated.

## Results

**Figure 1:**
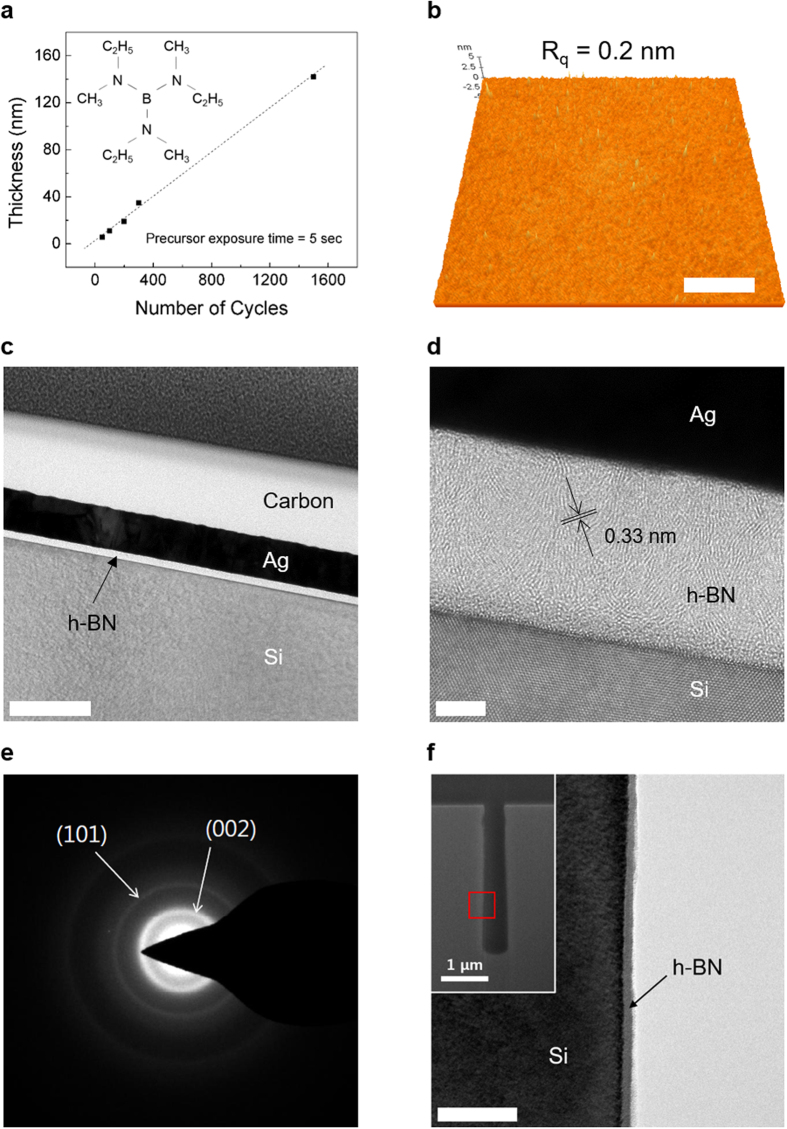
ALD of the h-BN films and their microscopic properties. (**a**) Film thickness as a function of the number of the ALD cycles. The inset shows the molecular structure of the precursor (TEMAB, C_9_H_24_BN_3_). (**b**) An AFM image of the h-BN film on a Si substrate. The scale bar corresponds to 1 μm. (**c**) A low-magnification TEM image of a uniform and flat h-BN film. (**d**) A high-magnification TEM image of the h-BN layered structure. The scale bars in (**c**) and (**d**) correspond to 200 nm and 5 nm, respectively. (**e**) An electron diffraction pattern of the h-BN film. (**f**) A TEM image of the h-BN film deposited on the trench sidewall. The scale bar corresponds to 100 nm. The inset shows the SEM cross-sectional image of the trench structure.

Tris(ethylmethylamino) borane (TEMAB, C_9_H_24_BN_3_) was used as a precursor for the h-BN synthesis (see the inset of Fig. 1a). The successful ALD was demonstrated by the obtained linear dependence of the growth characteristics on the number of deposition cycles (Fig. 1a). The h-BN growth rate was equal to 1.1 Å/cycle, while the precursor exposure time was maintained constant at 5 s. The saturation of the growth rate at longer precursor exposure times, a vapor pressure-temperature curve, and the results of the precursor thermogravimetric (TG) analysis are depicted in Supplementary Fig. 1a-c. Supplementary Fig. 1b shows that TEMAB has a sufficient vapor pressure (0.6 mbar at room temperature) to be used in ALD, while the TG curve in Supplementary Fig. 1c gradually decreases with temperature indicating that TEMAB is relatively stable and does not decompose until 200 °C (a negligible amount of TEMAB remained at temperatures above 200 °C, which indicated complete precursor evaporation in this temperature range). Also, ^1^H NMR spectrum of TEMAB shown in Supplementary Fig. 2 shows that the integration ratio (2:3:3) corresponds to the atomic structure of TEMAB as shown in the inset of Fig. 1a.

The atomic force microscopy (AFM) image depicted in Fig. 1b shows that the root mean square (R_rms_) value of the surface roughness for the as-grown h-BN film with a size of 5 μm × 5 μm is 0.2 nm. The surface flatness of h-BN was achieved due to the absence of a transfer process (required for CVD-grown h-BN films on metal foil), which usually produces wrinkles and polymer residues on the film surface. Furthermore, the resulting flat surface indicates the uniform distribution of precursor molecules on the substrate and the absence of residual species resulting from the reaction. The transmission electron microscopy (TEM) image obtained at low-magnification (Fig. 1c) also shows a highly flat surface with a uniform thickness. The high-magnification TEM image (Fig. 1d) displays the layered structure of the ALD-grown h-BN films indicating that the h-BN stacks are randomly distributed inside the film without any specific orientation. The nanoscale stacks are consistent with the low crystallinity revealed by the experimental analysis (described in the next section). The atomic structure ordering along the horizontal plane can potentially enhance the film quality and should be the focus of the future studies. The electron diffraction pattern shown in Fig. 1e contains ring-shaped patterns rather than points from the (002) and (101) planes. Therefore, we characterize the ALD-grown h-BN films as a nearly amorphous h-BN films with nanoscale stacks.

Conformal deposition of thin films is a key technology for wider applications in electronics and photonics. The conformality of thin film deposition can be evaluated by using a narrow trench structure. Owing to the surface-limited characteristic of ALD, the conformal deposition of h-BN was performed on a trench sidewall as shown in Fig. 1f (the inset displays the cross-sectional SEM image of the trench with a width of 500 nm and aspect ratio of 6:1). The TEM image depicted in Fig. 1f displays the cross-sectional image of the trench sidewall containing a uniformly deposited h-BN film, while its energy dispersive spectroscopy (EDS) mapping analysis is shown in Supplementary Fig. 3.

**Figure 2:**
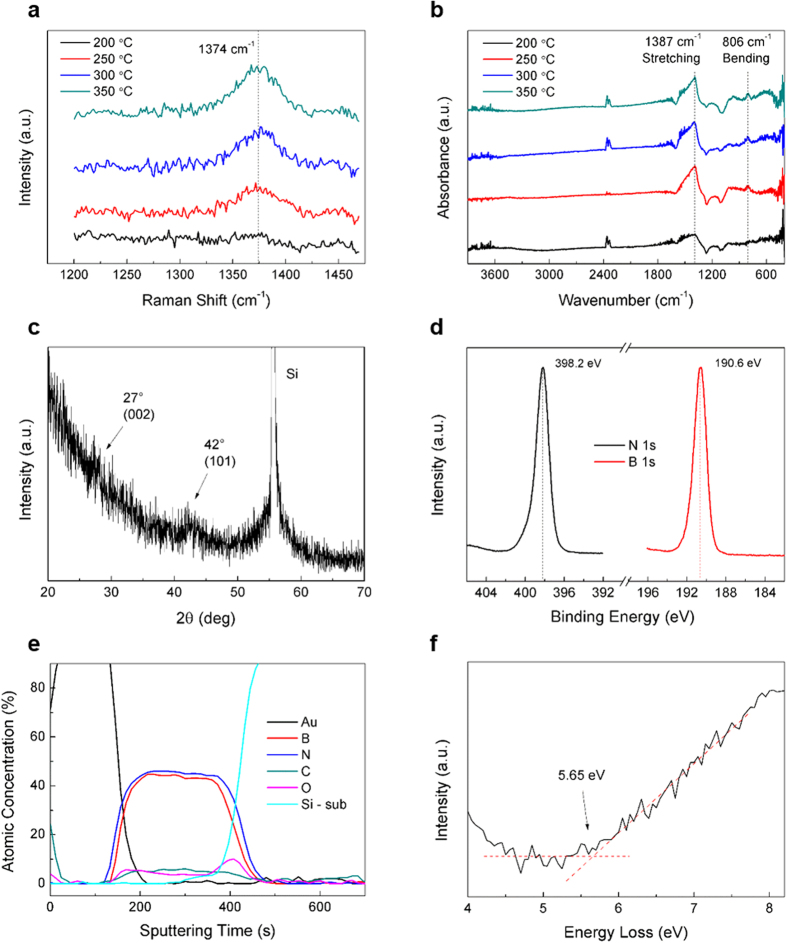
Characterization of the ALD-grown h-BN films. (**a**) Raman and (**b**) FT-IR spectra obtained at different film deposition temperatures. (**c**) An XRD spectrum for the h-BN film, which corresponds to the electron diffraction pattern presented in Fig. 1f. (d) XPS spectra for the B 1 s and N 1 s regions and (**e**) atomic concentrations obtained from the corresponding AES spectra, which confirm the 1:1 stoichiometric ratio between the B and N elements in the deposited films. (**f**) An EELS spectrum, characterized by a band-gap value of 5.65 eV. All films were grown at 300 °C and had thicknesses of 20 nm except for the XRD sample (100 nm).

The Raman spectra were obtained for h-BN deposited at four different temperatures: 200, 250, 300, and 350 °C (see Fig. 2a). The Raman peak was centered at 1374 cm^−1^ (which was close to the previously reported values for h-BN) and corresponded to the B-N E_2g_ in-plane vibrational mode, indicating the succesful formation of hexagonal BN species^3^,^4^. Moreover, the obtained Raman spectra clearly reveal that the temperature limit for hexagonal BN lies somewhere between 200 and 250 °C. The Fourier transform infrared spectroscopy (FT-IR) data presented in Fig. 2b produced a similar region of the temperature limit for hexagonal bonding, which was consistent with the Raman spectra. The peaks centered at 1387 cm^−1^ and 806 cm^−1^ correspond to the stretching and bending vibrations of h-BN, respectively. Fig. 2c shows the X-ray diffraction (XRD) spectrum containing the (002) and (101) peaks, which confirm the hexagonal atomic structure and are consistent with the electron diffraction pattern presented in Fig. 1f. The h-BN (002) and (101) peaks with small intensities centered at 27° and 42° indicate low crystallinity of the deposited h-BN films. From the (002) peak centered at 27° we calculated the interlayer gap using Bragg’s law ((2*dsinθ* = *λ, λ* = 1.54 Å (Cu K_α_), *θ* = 13.5°). The calculated interlayer gap of 0.33 nm is well consistent with TEM cross-section image in Fig. 1d, confirming the ordered structure of h-BN along the out-of-plane (002) direction. The experimental analysis suggested above show the peaks having broad width or low intensity which are considered to be from nearly amorphous characteristic of ALD-grown h-BN films with nanoscale stacks.

The B 1 s and N 1 s X-ray photoelectron spectroscopy (XPS) peaks are centered at 190.6 eV and 398.2 eV, respectively (Fig. 2d), and their positions are similar to those previously reported for h-BN^3^,^4^. The atomic ratio between the B and N elements calculated from the areas of the correspoding B 1 s and N 1 s spectra is equal to 0.94, which is close to the 1:1 stoichiometry of the h-BN film. The Auger electron spectroscopy (AES) measurements were performed to analyze the exact composition of the h-BN film (Fig. 2e). The estimated atomic ratio between the B and N species located below the Au passivation layer was equal to 0.96, which was consistent with the XPS results. In addition, small amounts of residual C (6 at.%) and O (4 at.%) elements were detected, which could be attributed to the adsorbed ligands originating from the precursor and contaminant species inside a vacuum chamber. Fig. 2f displays the low-loss region of electron energy loss spectroscopy (EELS) spectrum, which shows that the obtained h-BN band-gap value (5.65 eV) corresponds to that of a dielectric material with the insulating property and is comparable with the previous results of both theoretical and experimental studies (5.4–6.0 eV)^3^,^4^,^13^,^14^,^15^,^16^.

**Figure 3:**
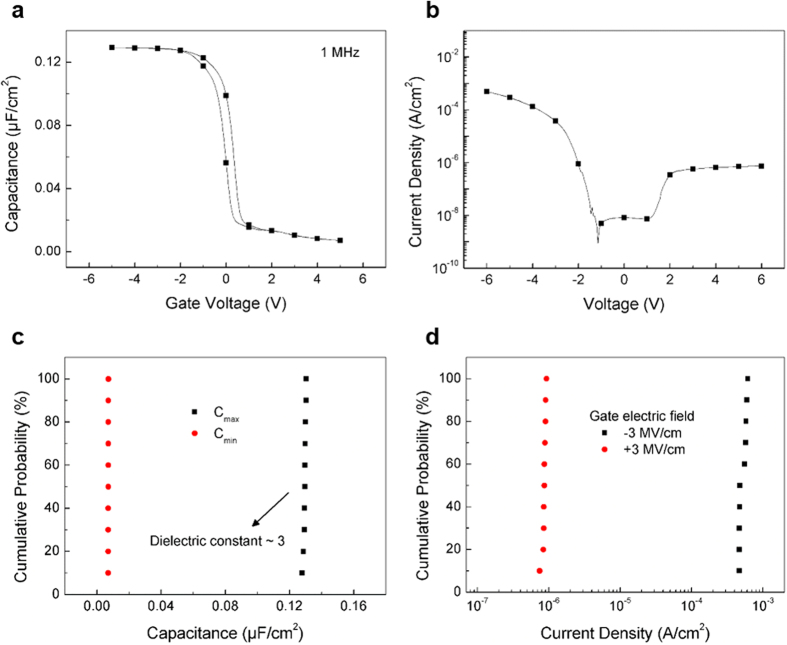
Electrical properties of the h-BN-containing MIS capacitors. (**a**) Capacitance-voltage and (**b**) leakage current density-voltage curves for the capacitors. Cumulative probabilities of the (**c**) maximum and minimum values of capacitance and (**d**) leakage current density measured at applied gate electric fields of −3 and + 3 MV·cm^−1^, respectively. All films were grown at 300 °C and had thicknesses of 20 nm unless specified otherwise.

The electrical performance of the PE-ALD grown h-BN films was evaluated by fabricating metal/h-BN/semiconductor capacitors (where Al was used as metal and p-type Si as semiconductor). Figure 3a shows that the capacitance-voltage (C-V) curves do not exhibit significant distortion or hysteresis, which indicates low levels of fixed or interface-trapped charge. The dielectric constant obtained from the maximum capacitance value was equal to 3, which was consistent with the previously reported dielectric constants for exfoliated (3–4)^17^ and CVD-grown h-BN (2–5)^18^,^19^. Fig. 3b shows the leakage current density-voltage (I-V) curves and the leakage current density measured at an applied electric field of −1 MV·cm^−1^ (10^−6^ A·cm^−2^), which is comparable with the previously reported values for CVD-grown h-BN^18^,^19^.

**Figure 4:**
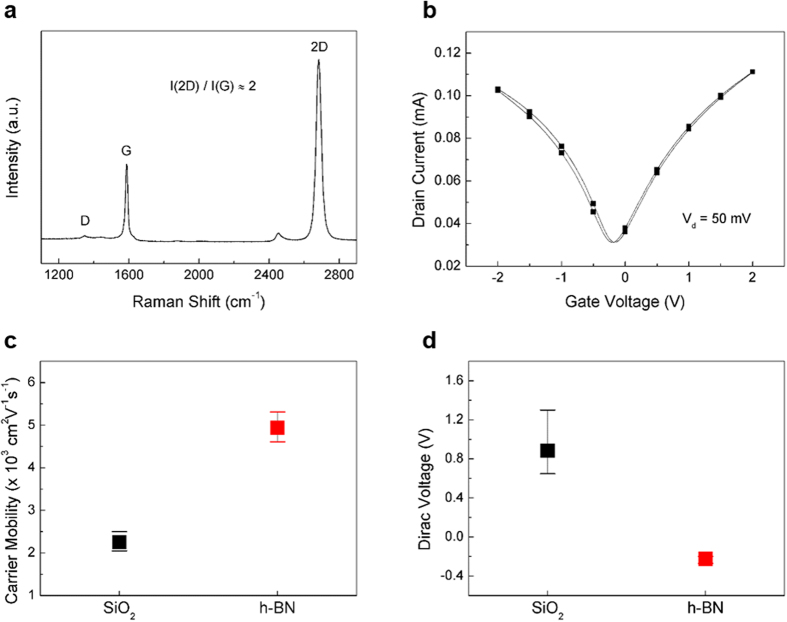
Characterization of the graphene field-effect transistors with h-BN and SiO_2_ substrates. (**a**) A Raman spectrum for graphene transferred on a SiO_2_ substrate. (**b**) A representative transfer curve for the GFET with an h-BN substrate. (**c**) Carrier mobility and (**d**) Dirac voltage distributions for the GFETs containing SiO_2_ and h-BN substrates. In all devices, the channel width and length were 40 μm and 15 μm, respectively.

Figures 3c and d show the cumulative probabilities of the maximum and minimum capacitance and the leakage current densities measured at gate electric fields of −3 and + 3 MV·cm^−1^, respectively. The obtained results exhibit very small variations of capacitances and leakage current densities among 10 different capacitors (below 4% on the log scale), indicating that highly uniform deposition of h-BN has been achieved due to the self-limiting nature of the ALD process, which is consistent with the saturated chemisorption of precursors at the surface of a deposition substrate.

h-BN has been considered as a suitable dielectric substrate for graphene-based electronic devices owing to its low density of charge impurities and small lattice mismatch with graphene (1.7%)^20^. Therefore, top-gate GFETs were fabricated to evaluate the substrate effect of h-BN on the electrical performance of graphene, which could be substantial considering the atomically small thickness of graphene^21^,^22^,^23^. The top-gate dielectric was Al oxide grown by Al evaporation followed by ALD. CVD-grown graphene on Cu foil was transferred by a poly(methyl methacrylate) (PMMA)-assisted wet transfer method, while metal electrodes were evaporated and patterned using photolithography and lift-off techniques. A schematic cross-sectional view of the top-gate GFET on h-BN substrate is depicted in Supplementary Fig. 4a. Figure 4a shows the Raman spectrum of transferred graphene, which confirms the successful graphene synthesis and transfer through the negligible D peak intensity and 2D/G intensity ratio of around 2. Figure 4b shows a representative transfer curve for the top-gate GFET fabricated on an h-BN substrate, which exhibits a typical V-shape without significant hysteresis. The electrical characteristic of the Al_2_O_3_ gate dielectric is provided in Supplementary Fig. 4b.

The carrier mobility values of graphene obtained from the transfer curves for 10 different transistors are shown in Fig. 4c. In order to estimate the graphene carrier mobility more accurately, we applied the fitting method in the form of the R_DS_-V_G_ plot presented in Supplementary Fig. 4c^24^,^25^,^26^. Hence, we were able to calculate the carrier mobility values without interference from the contact resistance and access resistance components. The obtained results exhibited a clear difference between the SiO_2_ and h-BN substrates (2,255 cm^2^·V^−1^·s^−1^ for SiO_2_ and 4,923 cm^2^·V^−1^·s^−1^ for h-BN). The observed enhancement of carrier mobility can be mainly attributed to the low density of charged impurities on the h-BN surface. Furthermore, Fig. 4d shows that the Dirac voltage (V_Dirac_) values measured for graphene on the SiO_2_ and h-BN substrates are equal to 0.9 V and −0.2 V, respectively. Thus, graphene on the h-BN substrate is characterized by a V_Dirac_ value close to 0 V, which indicates enhanced charge neutrality. Various adsorbates existing on the SiO_2_ surface (such as silanol (SiOH) groups) facilitate the adsorption of dipolar molecules and induce the p-type doping of graphene^21^,^27^,^28^,^29^,^30^. Therefore, Dirac voltage values for graphene on h-BN substrates close to 0 V indicate that the substrate surface is chemically inert and does not contain such adsorbed species.

## Conclusion

The large-scale synthesis of uniform h-BN films with controllable thicknesses was performed by PE-ALD at relatively low temperatures (250–350 °C). The synthesized h-BN species were characterized by various techniques such as AFM, TEM, Raman spectroscopy, and AES, and the obtained results were comparable with those previously reported for exfoliated and CVD-grown h-BN. The electrical properties of the synthesized h-BN correspond to a dielectric with a dielectric constant of 3 and low leakage current density. In addition, the electrical performance of graphene was enhanced when it was transferred on h-BN substrates. Therefore, the ALD technique can be utilized for the large-scale synthesis of highly uniform h-BN films for electronic applications.

## Methods

### Precursor (TEMAB, C_9_H_24_BN_3_) synthesis

Ethylmethylamine (C_3_H_9_N, 5.3 mol) and boron trichloride (BCl_3_, 0.85 mol) were dissolved in hexane (1000 mL), stirred at 0 °C for 3 h and at the room temperature for 4 h. Residual ethylmethylamine hydrochloride was filtered out and TEMAB was distilled under reduced pressure (34 °C, 1.46 Torr) for high purity (89% yield).

### PE-ALD process

We used a showerhead type ALD system (CN1 Co., Ltd., ATOMIC PREMIUM, Republic of Korea) with an 8 in stage. The temperatures of grown substrates were specified in the main text, while the precursor exposure time was fixed to 5 s (see Supplementary Fig. 1a). Ammonia plasma was introduced at a RF plasma power of 500 W for 7 s using a mixture of ammonia and N_2_ gases with flow rates of 240 and 60 sccm, respectively (chamber pressure: 0.5 Torr).

### Characterization

The TEM images were obtained by using a Cs-corrected Titan TEM instrument (FEI), while the Raman spectra were recorded by a high-resolution dispersive Raman spectrometer equipped with a 514 nm Ar ion laser source (ARAMIS, Horiba Jobin Yvon). ^1^H NMR spectrum was recorded on Bruker 400 MHz spectrometers. The capacitance-voltage curves were measured at ambient conditions using an Agilent 4284 A Precision LCR meter with an operational frequency of 1 MHz. The leakage current density values and transfer curves for the GFETs were obtained by using a chamber probe station (MST-1000B, MS Probe Station) and parameter analyzer (4200SCS, Keithley).

### Graphene synthesis

25 μm thick Cu foil (99.8% purity) was used as a substrate for graphene synthesis from a mixture of Ar, H_2_, and CH_4_ gases (with a flow rate ratio of 0.5:0.1:1), which was conducted at a temperature of 1000 °C and low pressure of around 500 mTorr for 1 h.

### Fabrication of the h-BN-containing capacitors and GFETs

The capacitors were fabricated by using an evaporated 200 nm thick Al layer as a top electrode. To manufacture GFETs, single-layer graphene was transferred onto an h-BN substrate by using the PMMA-assisted wet transfer method. ALD-deposited 24 nm thick Al oxide was used as a top gate dielectric layer. Au (60 nm)/Pd (15 nm) films were utilitzed as source and drain electrodes, and Au (60 nm)/Cr (5 nm) films were used as top electrodes (all electrodes in this study were deposited by thermal evaporation).

## Additional Information

**How to cite this article**: Park, H. *et al*. Large-scale synthesis of uniform hexagonal boron nitride films by plasma-enhanced atomic layer deposition. *Sci. Rep.* **7**, 40091; doi: 10.1038/srep40091 (2017).

**Publisher's note:** Springer Nature remains neutral with regard to jurisdictional claims in published maps and institutional affiliations.

## Supplementary Material

Supplementary Information
