# Supplementary material for: Large-scale synthesis of uniform hexagonal boron nitride films by plasma-enhanced atomic layer deposition
Source: Sci Rep. 2017 Jan 5;7:40091. doi: 10.1038/srep40091 (PMC5214515; doi:10.1038/srep40091)
Supplement: Supplementary Information [file srep40091-s1.pdf]

Supplementary Information

Large-scale synthesis of uniform hexagonal boron nitride films by plasma-enhanced atomic layer deposition

Hamin Park, Tae Keun Kim, Sung Woo Cho, Hong Seok Jang, Sang Ick Lee, and Sung-Yool Choi*

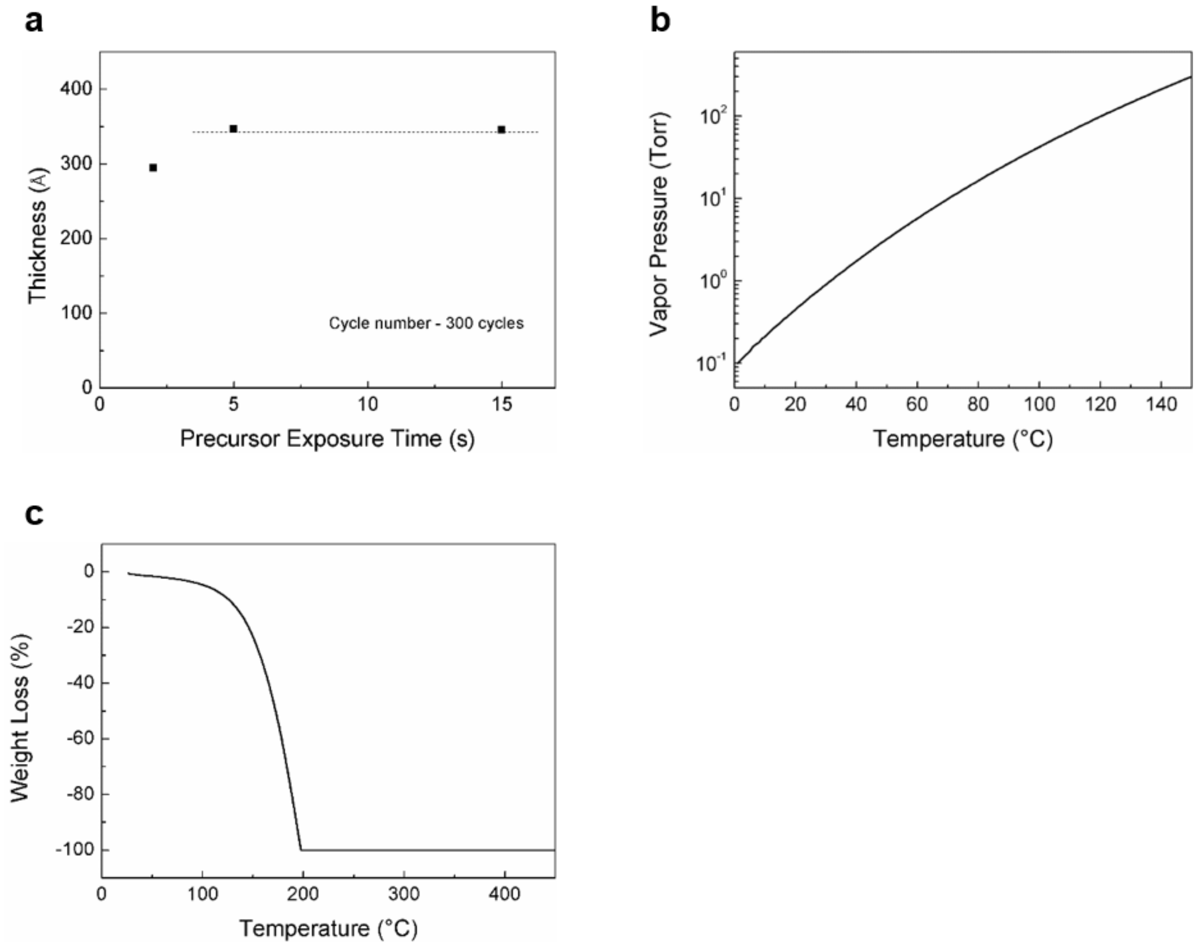

Supplementary Figure 1 | ALD precursor characteristics. (**a**) h-BN film thickness as a function of the precursor exposure time. (**b**) TEMAB vapor pressure as a function of temperature. (**c**) A TG curve recorded for the TEMAB precursor.

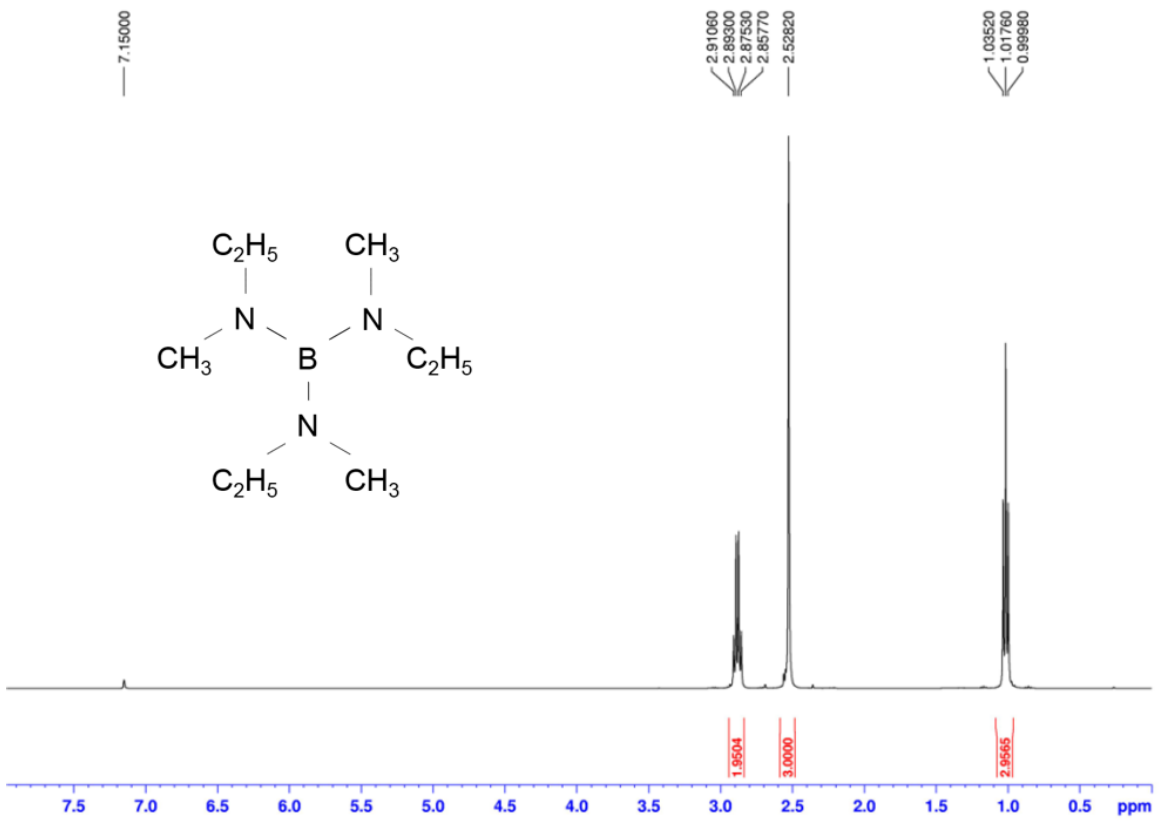

Supplementary Figure 2 | ^{1}H NMR spectrum for the precursor (TEMAB, $C_9H_{24}BN_3$). ^{1}H NMR (400MHz, C_6D_6): δ 2.88 (q, J = 7.1 Hz, 6H), 2.53 (s, 9H), 1.02 (t, J = 7.1 Hz, 9H).

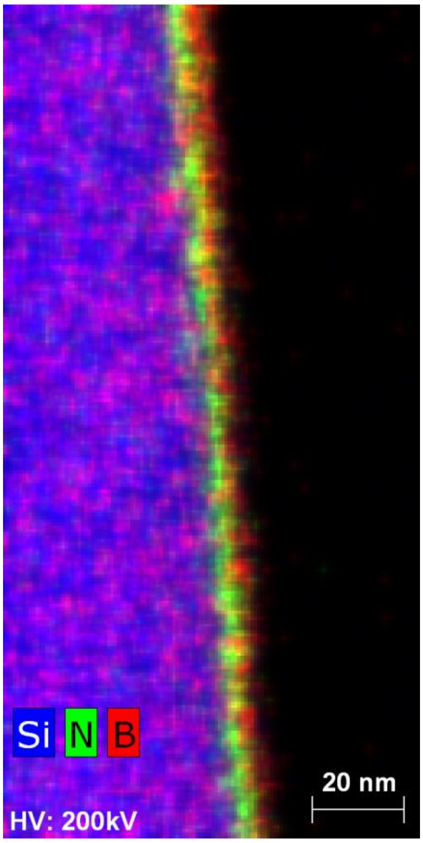

Supplementary Figure 3 | EDS elemental analysis. An EDS map for the cross-section of the h-BN film deposited on the trench sidewall.

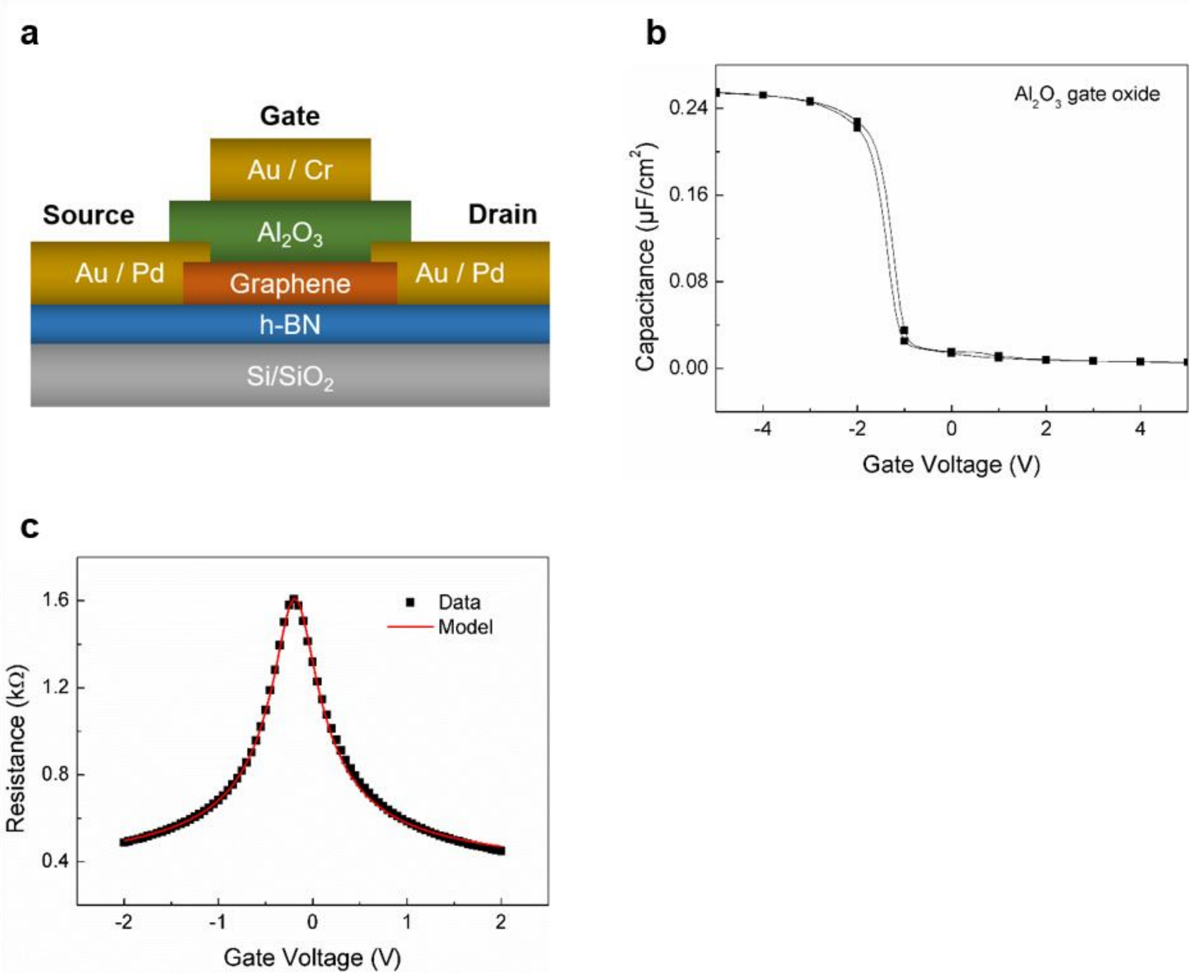

Supplementary Figure 4 | GFET characterization. (**a**) Schematic cross-sectional view of the top-gate GFET on h-BN substrate. (**b**) A capacitance-voltage curve for the Al_2O_3 gate dielectric film deposited on a Si substrate simultaneously with other graphene samples. A small positive charge was detected in the gate dielectric due to the presence of contaminant species in the deposition chamber. (**c**) A R_{DS}-V_G plot corresponding to the representative transfer curve depicted in Figure 4b. The fitted curve can be described by the following equation:

$$\mathrm{R} = 2R_C + 2R_A + \frac{L}{We\mu\sqrt{n_0^2 + n_G^2}}$$

where L and W are the gate length and width, and R_C and R_A are the contact and access resistances, respectively. n_0 is the residual carrier density at the Dirac voltage, and n_G is the gate-induced carrier density, which is equal to $C_{ox}(V_G-V_{Dirac})/e$.
